# Early aortic growth in acute descending aortic dissection

**DOI:** 10.1093/icvts/ivab351

**Published:** 2022-01-19

**Authors:** Mikolaj Berezowski, Johannes Scheumann, Friedhelm Beyersdorf, Marek Jasinski, Tomasz Plonek, Matthias Siepe, Martin Czerny, Bartosz Rylski

**Affiliations:** 1 Department of Cardiovascular Surgery, Faculty of Medicine, Heart Centre Freiburg University, University of Freiburg, Freiburg, Germany; 2 Department and Clinic of Cardiac Surgery, Wroclaw Medical University, Wroclaw, Poland; 3 Department Cardiac Surgery, Thorax Centrum Twente, Enschede, The Netherlands

**Keywords:** Type B aortic dissection, Aortic growth, Entry tear

## Abstract

**OBJECTIVES:**

Acute aortic dissection leads to the destabilization of the aortic wall, followed by an immediate increase in aortic diameter. It remains unclear how the aortic diameter changes during the dissection’s acute and subacute phases. The aim of this study was to evaluate the change in aortic geometry within 30 days after the onset of a descending aortic dissection.

**METHODS:**

Patients with acute type B and non-A non-B dissection who had at least 2 computed tomography angiography scans obtained within 30 days after the onset of dissection were evaluated. Exclusion criteria were a thrombosed false lumen, connective tissue disorders and endovascular or open aortic repair performed prior to the second computed tomography angiography.

**RESULTS:**

Among 190 patients with acute aortic dissection, 42 patients met our inclusion criteria. Their aortic geometry was analysed according to the computed tomography angiography scans obtained between 0–3 (*N* = 35), 4–7 (*N* = 9) and 8–30 (*N* = 12) days after the dissection onset. The highest aortic diameter growth rate was observed in the first quartile of the thoracic aorta and measured 0.66 (0.06; 1.03), 0.29 (−0.01; 0.41) and 0.06 (−0.13; 0.26) mm/day at 0–3, 4–7 and 8–30 days after the dissection, respectively. Proximal entry location (*P* = 0.037) and entry located at the arch concavity (*P* = 0.008) were associated with a higher aortic diameter increase.

**CONCLUSIONS:**

Early rapid growth occurs during the first week after the descending aortic dissection—most intensely over the first 3 days, and this is associated with the location of the dissection’s entry.

**Figure:**
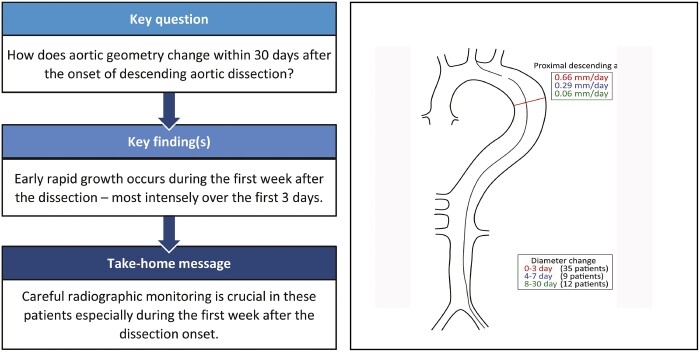

## INTRODUCTION

Acute aortic dissection leads to the destabilization of the aortic wall, followed by an immediate increase in aortic diameter [1, 2], which tends to expand during the dissection’s acute phase. About 4 weeks after the dissection, aortic growth stabilizes, plateauing 3 months later [3].

Thoracic endovascular aortic repair (TEVAR) is the treatment of choice in complicated acute type B aortic dissection [4]. One of the parameters included in the definition of complicated type B dissection is early aortic expansion [4]. It is well known in clinical practice, especially in the acute phase of dissection, when the rapidly increasing aortic size leads to aortic rupture. This underlines the need for predictors to identify patients at higher risk. Currently, there is no clear-cut definition of early aortic expansion in acute descending aortic dissection in the literature.

It remains unclear how rapidly the aortic diameter changes during the acute and early subacute phases of dissection. The aim of this study was to evaluate the change in aortic geometry within 30 days after the onset of descending aortic dissection.

## MATERIALS AND METHODS

### Study population

Between 2003 and 2017, 190 patients with acute type B and non-A non-B (type B with dissection components in the aortic arch [5]) aortic dissection were admitted to our clinic. Of the 177 patients who had at least 2 computed tomography angiography (CTA) scans taken within 30 days after the onset of dissection, we excluded those with poor imaging quality, thrombosed false lumen, connective tissue disorders and endovascular or open aortic repair performed prior to the second CTA. The time of aortic dissection was estimated based on the time of the onset of the symptoms obtained from the medical history. This information was available in all patients included in the final analysis.

### Ethics statement

Our institutional review committee approved this retrospective study, and the need for informed consent was waived.

### Protocol of treatment

Patients with diagnosed aortic dissection are transferred to the intensive care unit after hospital admission. In case of complicated dissection when emergency intervention is required they are naturally moved to the operating theatre as quickly as possible. All other patients are monitored in the intensive care unit usually for 3–5 days and then they are transferred to intermediate care. They have blood pressure monitored with systolic blood pressure target values of <130 mmHg and receive pain medications. CTA examinations are routinely taken at the admission, 24 h, 72 h, a week after dissection onset and before the discharge. High-risk patients with complicated dissection are considered those with refractory pain or hypertension, patients with radiographic features such as arch entry non-A non-B aortic dissection, type B dissection with the most proximal entry tear within the aortic arch concavity, maximal thoracic aortic diameter >40 mm, ongoing aortic expansion of >1 mm/day. All of them are evaluated carefully and scheduled for the early urgent intervention. Currently, the majority of uncomplicated type B dissections are managed endovascularly within the subacute phase i.e. 6 weeks—3months, while arch entry non-A non-B dissection undergo urgent total arch replacement with the frozen elephant trunk technique.

### Image analysis

Digital Imaging and Communications in Medicine (DICOM) data were analysed using 3mensio Vascular Version 7.2 (3mensio Pie Medical Imaging BV, Maastricht, Netherlands). All of the included patients’ CTA scans were electrocardiography gated. A slice thickness of 1 mm or less was accepted.

A centreline was created from the aortic valve annulus to the aortic bifurcation. The thoracic descending aorta was defined as starting at the distal edge of the left subclavian artery (LSCA) until the coeliac trunk ostium and divided into 4 segments of equal length by appropriate planes perpendicular to the centre line. Planimetric measurements yielding luminal area and maximum and minimum diameters via semi-automated polygonal border tracing were obtained by contour tracking at the following planes perpendicular to the centreline: the aortic arch between the left carotid and LSCA (LSCA; only for non-A non-B dissections), at the LSCA, at the level of the most proximal entry, the thoracic descending aorta at the first, second and third quartiles, at the coeliac trunk and mid-abdominal aorta between the renal arteries and aortic bifurcation. All reported diameters are diameters calculated according to the cross-sectional areas if not indicated otherwise. Minimum and maximum diameters were obtained to calculate the aortic ellipticity index, defined as the maximum diameter divided by minimal diameter, which was calculated for each plane. Circularity was defined as an ellipticity index <1.1. Geometric changes were measured only in those segments involved in the dissection process. Aortic growth was calculated as a difference between measurements obtained at different time points contained within the following time intervals: 0–3, 4–7, 8–30 days, and divided by the time between these measurements expressed in days. Rapid aortic diameter growth was defined as growth of at least 1 mm/day. The most proximal entry location was assessed according to the aortic segment, orientation towards arch convexity (outer curvature) or concavity (inner curvature) and distance to the LSCA. We considered an entry tear in the aortic arch as an entry with 0 mm distance to the LSCA. Entry tear size was assessed as the maximal diameter of the tear in the multiplanar reconstruction. Large entry tear was considered as >10 mm.

Volume was assessed in each aortic segment as follows: (i) the aortic arch beginning immediately proximal to the origin of the innominate artery and extending to the plane immediately distal to the origin of the LSCA (only for non-A non-B dissections); (ii) the proximal descending thoracic aorta beginning at the plane immediately distal to the LSCA’s origin and extending to the plane immediately proximal to the coeliac trunk and (iii) the abdominal aorta beginning at the plane immediately proximal to the coeliac trunk and extending to the aortic bifurcation. Volumetric measurements were taken semi-automatically by marking the aorta’s outer border every 22.5° craniocaudal direction starting at the corresponding proximal and ending at the distal plane defining the analysed aortic segment. Each segment’s total volume was computed by referring to the aortic wall’s outer surface. Volume was measured only in segments involved in the dissection process.

### Statistical analysis

Continuous data are reported as median (first quartile; third quartile), while categorical variables are shown as counts, percentages. Independent samples *t*-test was applied when compared continuous data. The Mann–Whitney rank-sum test was employed in case of not normally distributed variables. The normality of the quantitative variables was tested using the Shapiro–Wilk test. In case of small group sizes (*n* < 5), Fisher’s exact test was used. Due to the limited study cohort, the statistical analysis was performed only for the 0–3 days group. Time interval classification is arbitrary and is based on authors clinical practice and on the preliminary analysis showing the rate of aortic growth during the time. Two-sided *P*-values were computed, and a difference was considered statistically significant at *P *<* *0.05. All statistical calculations were made using SigmaPlot 12.3 (Systat Software, San Jose, CA, USA).

## RESULTS

**Figure 1:**
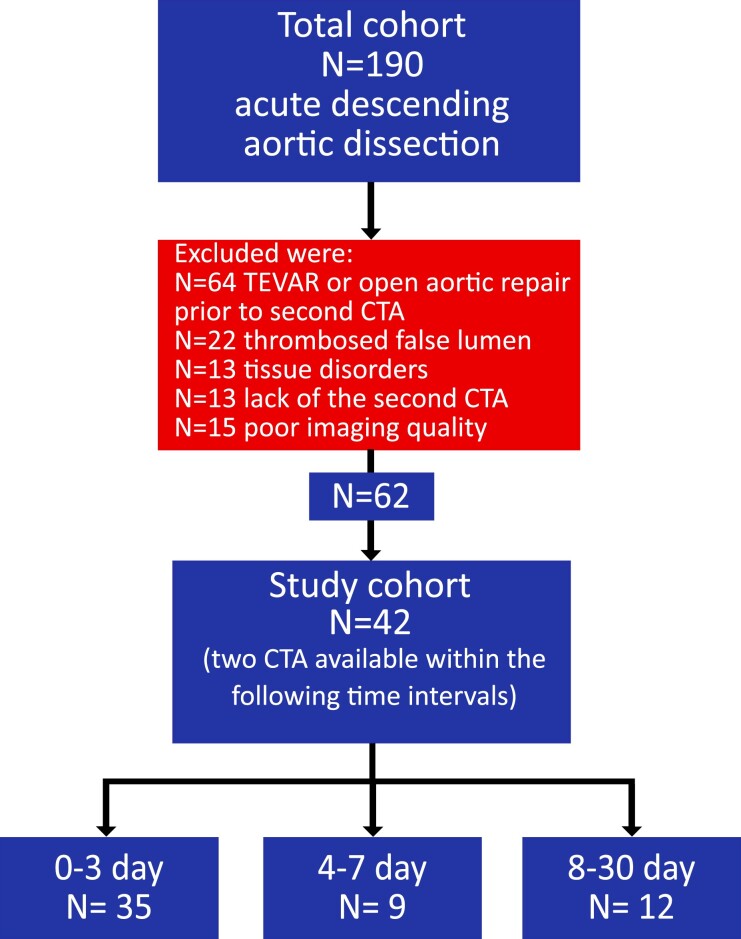
Flow of patient selection process.

Overall, enrolled were 42 patients [median age: 66 (first quartile: 55; third quartile: 72) years; 74% men] who could be classified to at least one of these time intervals groups: 0–3 (*N* = 35), 4–7 (*N* = 9) and 8–30 days (*N* = 12) (Fig. 1). Eleven of 42 patients met the inclusion criteria of 2 time intervals groups and 1 patient met the criteria of 3 groups. Seven of 42 had their initial CTA scan done at least 3 days after the onset of symptoms and did not meet the criteria of 0–3 days group.

Fifteen (36%) of 42 patients suffered from acute non-A non-B aortic dissection, 27 (64%) from acute type B dissection.

Clinical and demographic data are summarized in Table 1. Among 42 patients included in this analysis, 18 required aortic repair within 30 days after dissection onset. Indications for endovascular or surgical aortic repair are illustrated in Table 2.

**Table 1:** Clinical data

Parameters	*N* = 42
Demographics	
Age (years)	66 (55; 72)
Male gender	31 (74%)
Risk factors	
Hypertension	39 (93%)
Dyslipidaemia	13 (31%)
CAD	3 (7%)
COPD	2 (5%)
Renal failure	3 (7%)
Diabetes	2 (5%)
Smoking history	13 (31%)
Previous cardiac surgery	2 (5%)
Dissection aetiology	
Spontaneous	41 (98%)
Iatrogenic	1 (2%)
Treatment within 30 days	
Conservative	24 (57%)
TEVAR	17 (41%)
Open surgical	1 (2%)

Continuous values are medians (first quartile; third quartile), categorical values are *n* (%).

CAD: coronary heart disease; COPD: chronic obstructive pulmonary disease; TEVAR: thoracic endovascular aortic repair.

**Table 2:** Indications for aortic repair

Indication	TEVAR	Open repair
	*N* = 17	*N* = 1
Early aortic expansion	1 (6%)	0
Aortic rupture	1 (6%)	0
Dissection progression into the aortic arch	1 (6%)	1 (100%)
Malperfusion	2 (12%)	0
Recurrent of refractory pain	6 (35%)	0
Refractory hypertension	2 (12%)	0
True lumen collapse	3 (17%)	0
Penetrating aortic ulcer	1 (6%)	0

Categorical values are *n* (%).

TEVAR: thoracic endovascular aortic repair.

### Initial aortic diameter, area and ellipticity

Overall median aortic diameters measured at the initial CTA 37.2 (33.1; 39.3), 37.9 (34.2; 41.4) and 35.2 (32.4; 39.6) mm at the LSCA, first and second quartiles of the thoracic aorta, respectively. The greatest aortic diameter was measured at the level of the most proximal entry tear [40.2 (35.8; 43.6) mm], the smallest at the aortic bifurcation [23.3 (22; 26.3) mm]. Initial aortic diameters and areas are summarized in Table 3.

**Table 3:** Initial aortic diameter and area at the first computed tomography angiography after the admission.

Aortic level	Diameter (mm)	Area (mm^2^)
LSCA (*n* = 39)	37.2 (33.1; 39.3)	1086.3 (857.5; 1212.4)
Most proximal entry tear (*n* = 42)	40.2 (35.8; 43.6)	1265.5 (1006.1; 1488.9)
Thoracic aorta first quartile (*n* = 40)	37.9 (34.2; 41.4)	1124.6 (919.5; 1345.5)
Thoracic aorta second quartile (*n* = 41)	35.2 (32.4; 39.6)	972.6 (824.1; 1231)
Thoracic aorta third quartile (*n* = 41)	33 (31.5; 38.4)	854.9 (778.9; 1157.5)
Coeliac trunk (*n* = 39)	33.3 (30; 36.5)	870.5 (706.5; 1043)
Mid-abdominal aorta (*n* = 34)	24.2 (22.9; 27)	448.4 (402.7; 565.9)
Aortic bifurcation (*n* = 28)	23.3 (22; 26.3)	411.7 (379.9; 538.9)

Continuous values are medians (first quartile; third quartile) and are given in mm (diameter) or mm^2^ (area). The diameter and the area are those obtained on the initial CTA and pertain only dissected aortic segments.

CTA: computed tomography angiography; LSCA: left subclavian artery.

The highest median aortic ellipticity index was calculated at the LSCA [1.09 (1.07; 1.13)], the lowest at the mid-abdominal aorta [1.06 (1.04; 1.9)].

### Most proximal entry tear and extension of the dissection

Six (14%) of 42 patients had their most proximal entry tear in the aortic arch, 25 (60%) between LSCA and the first quartile of the thoracic aorta, 9 (21%) between the first quartile and coeliac trunk and 2 (5%) in the abdominal aorta. Of the 31 patients with most proximal entry in the aortic arch or between LSCA and the first quartile of the thoracic aorta, 20 had the entry located at the convexity, 7 at the concavity of the distal aortic arch; the entry’s location in 4 patients was not definitively assessable. Median LSCA—most proximal entry tear distance was 27 (5.8; 99.8) mm. The median size of the entry tear was 8 (6.0; 12.0) mm. Sixteen (38%) patients had a large entry tear (>10 mm).

In 40 (95%) patients, the dissection extended proximally to at least the first quartile of the thoracic descending aorta. All except one patient (98%) presented an aorta dissected distally to at least the mid-abdominal aorta.

### Diameter and area change

**Figure 2:**
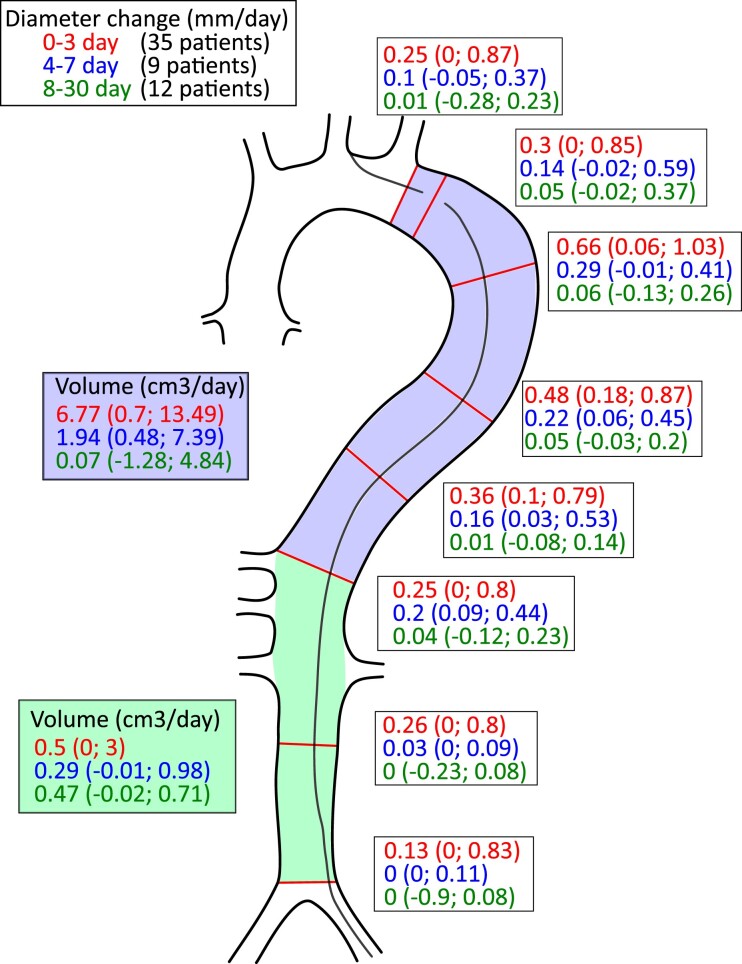
Aortic diameter and volume changes within 0–3, 4–7, 8–30 days after the acute type B dissection onset. Data are presented as median (first quartile; third quartile) and given in mm for diameter or cm^3^ for volume. CTA: computed tomography angiography; TEVAR: thoracic endovascular aortic repair.

The greatest aortic growth was observed between 0–3, and the lowest between 8–30 days after the dissection onset (Fig. 2). Aortic growth was faster in the descending thoracic aorta’s proximal portion than in the other more distal segments. The greatest aortic growth of 0.66 (0.06; 1.08) mm/day was measured at the first quartile of the thoracic aorta between 0 and 3 days, slowing down to 0.29 (-0.01; 0.41) mm/day between 4 and 7 days and to 0.06 (-0.13; 0.26) between 8 and 30 days.

Area changes corresponded with the diameter changes for 0–3, 4–7 and 8–30 days groups and are summarized in Table 4.

**Table 4:** Area change

Aortic level	0–3 day	4–7 day	8–30 day
	(*N* = 35)	(*N* = 9)	(*N* = 12)
	mm^2^/day	mm^2^/day	mm^2^/day
LSCA	13.4 (0; 53.1)	6.6 (-2.7; 21.9)	0.7 (-14; 13.1)
Most proximal entry tear	20.1 (0; 54.2)	6.6 (-1.3; 36.5)	3.3 (-1.3; 24.6)
Thoracic aorta first quartile	34 (3.3; 59.4)	16.5 (-0.4; 24.8)	4 (-8.2; 17.3)
Thoracic aorta second quartile	26 (7.9; 48)	12.3 (3.4; 23.2)	2.5 (-1.8; 13)
Thoracic aorta third quartile	21.7 (5.9; 37.3)	9.6 (1.7; 26.9)	0.5 (-4.1; 7.5)
Coeliac trunk	12.9 (0; 37.7)	10.5 (3.9; 25.7)	1.7 (-7.5; 13.1)
Mid-abdominal aorta	8.7 (0; 28.5)	1.1 (0; 4)	0 (-9.2; 3.2)
Aortic bifurcation	4.9 (0; 29)	0 (0; 4.2)	0 (-3.3; 3.1)

Continuous values are medians (first quartile; third quartile) and are given in mm^2^/day. Area change was calculated only in aortic segments involved in dissection process.

LSCA: left subclavian artery.

### Aortic growth within first 3 days in relation to entry site and aortic diameter

Among 35 patients in the 0–3 day group, 18 (51%) developed aortic growth of at least 1 mm/day at the level of LSCA or along the thoracic aorta. Twenty-five patients with the most proximal entry tear located in the aortic arch or between LSCA and the first quartile of the thoracic aorta had higher aortic growth rate when compared to the rest [1.3 (0.9; 1.9) mm vs 0.7 (0.5; 1.7) mm, *P* = 0.037]. All of the 7 patients whose most proximal entry was at the distal aortic arch concavity developed aortic growth of at least 1 mm/day (*P* = 0.008). All of the 6 patients whose most proximal entry was located in the aortic arch revealed aortic growth measuring 1 mm/day or more (*P* = 0.019). There was no correlation between the most proximal entry tear size was and aortic growth of 1 mm/day or more.

There was no difference in aortic growth rate between 11 patients who had maximal aortic diameter ≥45 mm and the others [1.1 (0.7; 1.4) mm vs 1.3 (0.9; 2.0), *P* = 0.181]. Nine of 18 (50%) patients who exhibited early aortic growth of at least 1 mm/day were managed conservatively, versus 12 of 17 (71%) with <1 mm/day aortic growth (*P* = 0.294).

### Volume change

Median volume of the descending thoracic aorta and abdominal aorta at the most proximal CTA were 265.4 (215.9; 352.3) and 80.1 (61.7; 106.3) cm^3^, respectively. Descending thoracic aorta volume increased by 6.8 (0.7; 18.5), 1.9 (0.5; 7.4), 0.1 (-1.3; 4.8) cm^3^/day in the 0–3, 4–7 and 8–30 day groups, respectively (Fig. 2).

### Aortic arch geometry change in non-A non-B dissection

Among 15 patients with acute non-A non-B aortic dissection, we assessed 13 in the 0–3, 4 in the 4–7 and 4 in the 8–30 day groups. Mid-arch diameter, area and aortic arch volume at the initial CTA were 34.8 (33.3; 38.5) mm and 947.6 (867.7; 1157.4) mm^2^ 57.3 (42.5; 64.6) cm^3^, accordingly. The change in mid-arch diameter measured 0.15 (-0.02; 0.39), 0.02 (0; 0.07), -0.13 (-0.32; 0.19) mm/day in the 0–3, 4–7 and 8–30 day group, respectively. Aortic arch volume changes amounted to 0 (-0.12; 2.03), 0.11 (0.03; 0.27), -0.2 (-0.24; 0.63) cm^3^/day within 0–3, 4–7 and 8–30 days, accordingly.

### Aortic rupture

We documented an aortic rupture in a patient with an initial diameter of 30.8 mm at the second quartile of the thoracic aorta and the highest aortic growth rate of 3 mm/day measured at the descending aortic first quartile. His aorta ruptured 1 day after the dissection onset between the second and the third quartiles of the thoracic aorta, increasing its diameter from 30.3 to 44.1 mm at the rupture level. Descending thoracic aorta volume increased from 187.3 to 244.3 cm^3^ (+30%). The dissection’s most proximal entry tear was located 40 mm distally to the LSCA.

## DISCUSSION

It is important to assess the changes in aortic anatomy and the patient’s physical condition during the first days after the onset acute aortic dissection to determine whether surgical, endovascular or conservative treatment is best. It remains unclear how rapidly the aorta expands in the very first days after the dissection onset. It was this study’s aim to evaluate aortic geometry changes within 30 days after the dissection.

Our study findings of can be summarized as follows:

- Acute aortic dissection leads to aortic wall destabilization and continuous aortic diameter growth in the acute and subacute phase.
- The greatest aortic growth occurs within the first week after the dissection, being most intense during the first 3 days.
- Patients presenting rapid aortic growth (≥1 mm/day) during the first 3 days after the dissection revealed their most proximal entry tear more proximally to the LSCA or in the aortic arch and at the distal aortic concavity when compared to patients whose aortic growth was <1 mm/day.
- The greatest descending aortic expansion was measured in the proximal segments.

### Relationship to previous studies

The vast majority of studies [6–8] addressing aortic diameter and type B aortic dissection assessed aortic growth in the chronic phase. One investigation reported that acute aortic dissection leads to an immediate mid-descending thoracic aortic diameter increase averaging 23% [2]. After the dissection, the aorta dilates over time. The greatest aortic growth occurs in the acute phase, decreasing during the subacute phase and reaching a plateau in the chronic phase [3]. However, until now exact aortic early expansion remained unclear.

In the recent systematic review by Spinelli *et al.* [9] on the predictors of aortic growth in acute type B aortic dissection, the authors have noticed the results are controversial and conflicting. The most documented positive predictor was aortic size at admission, which was not confirmed in our study. However, most of these studies focused on the long-term evaluation, contrary to our investigation evaluating the very early phase of acute descending aortic dissection.

### Early rapid aortic growth

Our results show that early rapid aortic growth generally applies to the first week after acute descending aortic dissection. The greatest aortic diameter increase occurred during the first 3 days after the dissection onset and then gradually decreased with the time. The first half of the acute phase seems to be the riskiest time, when dissected aortic tissue is exceptionally fragile. It is well known that TEVAR in this early phase is associated with higher risk of aortic rupture and retrograde type A aortic dissection. The risk of these complications decreases in the subacute phase. The aorta becomes less vulnerable to rupture, remaining able to benefit from the positive aortic remodelling process induced by TEVAR [10, 11].

### Most proximal entry tear location and rapid aortic growth

We found that patients who revealed rapid aortic growth within the first 3 days had their most proximal entry tear more proximally to the LSCA or in the aortic arch and usually at the inner curvature. An entry location at the distal arch concavity or proximal to the LSCA is associated with poorer outcomes following type B aortic dissection and with a high risk for adverse events such as early aortic expansion, malperfusion and rupture [12, 13]. The rapid aortic growth observed in these patients in our series is another reason for careful radiographic monitoring in the dissection acute phase.

### The upper descending aorta grows faster

The greatest aortic growth of 0.66 mm/day was measured at the first quartile of the thoracic aorta within first 3 days after the dissection. Similar tendency with the highest diameter increase in the upper descending aortic segment has been observed in the study investigating immediate changes after the onset of acute type B aortic dissection [2]. The present findings demonstrate that this pattern of early aortic geometry changes resembles that observed immediately after dissection onset.

### Limitations

This study is limited by the low number of patients we enrolled. The exact time of the aortic dissection onset based on the symptoms’ onset (i.e., pain) might not coincide with the actual dissection in all patients. True and false lumen diameter changes were not provided in this study. However, we hypothesis the dynamic changes of true and false lumen diameters are dependent on many factors such as cardiac cycle and the moment of scan slice taken at the certain level of the aorta. Thus they role as a reliable predictors of aortic growth in the early acute phase of dissection might be limited. We enrolled patients with type non-A non-B aortic dissection what could bias the results. However, the course is very similar to patients with type B aortic dissection with entry tear proximal to the aortic arch or localized at the arch concavity. We excluded patients with thrombosed false lumen as it is a well-documented negative predictor of aortic growth and could disturb the homogeneity of the study cohort. Excluding many patients due to TEVAR or open aortic repair prior to the second CTA may have biased our results. Since we observed only 1 aortic rupture in this series, our dataset does not allow us to make definitive conclusions regarding the aortic growth rate-threshold associated with a high risk of aortic rupture.

## CONCLUSIONS

Early, relevant aortic growth occurs within the first week after a descending aortic dissection, becoming most intense during the first 3 days. Exhibiting the most proximal entry tear located in the aortic arch or in the proximal descending aorta, especially at the aortic concavity, is associated with rapid early aortic expansion. More investigation is necessary to identify the threshold aortic growth rate associated with risk of aortic rupture and to confirm the findings of our study.

**Conflict of interest:** none declared. 

### Author contributions

**Mikolaj Berezowski:** Conceptualization; Data curation; Formal analysis; Investigation; Methodology; Visualization; Writing—original draft. **Johannes Scheumann:** Formal analysis; Methodology; Validation; Writing—review & editing. **Friedhelm Beyersdorf:** Conceptualization; Formal analysis; Validation; Writing—review & editing. **Marek Jasinski:** Conceptualization; Formal analysis; Validation; Writing—review &editing. **Tomasz Plonek:** Conceptualization; Formal analysis; Validation; Writing—review & editing. **Matthias Siepe:** Conceptualization; Formal analysis; Validation; Writing—review & editing. **Martin Czerny:** Conceptualization; Formal analysis; Validation; Writing—review & editing. **Bartosz Rylski:** Conceptualization; Formal analysis; Methodology; Project administration; Supervision; Validation; Writing—review & editing.

### Reviewer information

Interactive CardioVascular and Thoracic Surgery thanks Diana Reser and the other anonymous reviewers for their contribution to the peer review process of this article.

## ABBREVIATIONS

CTA: Computed tomography angiography
LSCA: Left subclavian artery
TEVAR: Thoracic endovascular aortic repair
